# Clinical feasibility of umbilical cord tissue-derived mesenchymal stem cells in the treatment of multiple sclerosis

**DOI:** 10.1186/s12967-018-1433-7

**Published:** 2018-03-09

**Authors:** Neil H. Riordan, Isabela Morales, Giselle Fernández, Nicole Allen, Neal E. Fearnot, Michael E. Leckrone, Dedra Jones Markovich, Darla Mansfield, Dorita Avila, Amit N. Patel, Santosh Kesari, Jorge Paz Rodriguez

**Affiliations:** 1 Stem Cell Institute, Panama City, Panama; 2 MediStem Panama Inc., Clayton, City of Knowledge, Panama City, Panama; 3 Cook Advanced Technologies, West Lafayette, IN USA; 4 Cook Medical Technologies, Bloomington, IN USA; 5 Department of Surgery, University of Miami School of Medicine, Miami, FL USA; 6 Department of Translational Neurosciences and Neurotherapeutics, John Wayne Cancer Institute and Pacific Neuroscience Institute, Santa Monica, CA USA

**Keywords:** Multiple sclerosis, Umbilical cord stem cells, Stem cell therapy, Multiple sclerosis treatment, Mesenchymal stem cells

## Abstract

**Background:**

Multiple sclerosis (MS) is a progressively debilitating neurological condition in which the immune system abnormally erodes the myelin sheath insulating the nerves. Mesenchymal stem cells (MSC) have been used in the last decade to safely treat certain immune and inflammatory conditions.

**Methods:**

A safety and feasibility study was completed on the use of umbilical cord MSC (UCMSC) as a treatment for MS. In this 1-year study, consenting subjects received seven intravenous infusions of 20 × 10^6^ UCMSC over 7 days. Efficacy was assessed at baseline, 1 month and 1 year after treatment, including magnetic resonance imaging (MRI) scans, Kurtzke Expanded Disability Status Scale (EDSS), Scripps Neurological Rating Scale, Nine-Hole Peg Test, 25-Foot Walk Test, and RAND Short Form-36 quality of life questionnaire.

**Results:**

Twenty subjects were enrolled in this study. No serious adverse events were reported. Of the mild AEs denoted as possibly related to treatment, most were headache or fatigue. Symptom improvements were most notable 1 month after treatment. Improvements were seen in EDSS scores (p < 0.03), as well as in bladder, bowel, and sexual dysfunction (p < 0.01), in non-dominant hand average scores (p < 0.01), in walk times (p < 0.02) and general perspective of a positive health change and improved quality of life. MRI scans of the brain and the cervical spinal cord showed inactive lesions in 15/18 (83.3%) subjects after 1 year.

**Conclusions:**

Treatment with UCMSC intravenous infusions for subjects with MS is safe, and potential therapeutic benefits should be further investigated.

*Trial registration* ClinicalTrials.gov NCT02034188. Registered Jan 13, 2014. https://clinicaltrials.gov/ct2/show/NCT02034188

## Background

Multiple sclerosis (MS) is an immune-mediated inflammatory disease in which the immune system progressively destroys its own myelinated axons in the central nervous system, in episodes lasting from a few months to many years in duration. The eventual demyelination and axonal degeneration can cause serious and debilitating motor, sensory, balance and cognitive problems, disability, serious complications, and negatively impact quality of life [1–3].

While there is no known cure for MS, up to 82% of costs incurred by MS patients are spent on drugs [4]. Treatments available include steroids for temporary flare-ups, disease-modifying drugs, and drugs targeting specific symptoms. While these may reduce the frequency of exacerbations and slow disease progression, none have myelin or nerve regenerative capability to restore the cumulative damage already in place [5].

Mesenchymal stem cells (MSC) derived from bone marrow, adipose, or other sources can exert inhibitory effects on immune-mediated disease states [6–10]. In particular, MSC derived from umbilical cord (UC) Wharton’s Jelly possess a high proliferative and expansive ability, an enhanced therapeutic activity compared to other MSC [11–14], and superior production of growth factors that stimulate secretions responsible for therapeutic potential [15].

The safety of MSC therapy for MS has been demonstrated in several trials [16–20]. We reported three subjects treated with MSC and stromal vascular fraction with no adverse effects; all showed clinical improvements in cognitive and motor function and presented no new lesions on magnetic resonance imaging (MRI) [21]. More recently, trials with placenta-derived MSC [22], or with intravenous UCMSC [23] reported few mild or moderate adverse effects, as well as some improvement in Expanded Disability Status Scale (EDSS) scores.

In this study, we sought to determine the safety and the efficacy of allogeneic UCMSC treatment in subjects with MS.

## Methods

This open-label, single-arm, single-center phase 1/2 study was designed to assess the safety and efficacy of the intravenous administration of UCMSC for the treatment of MS. The study was approved by the Panamanian Institutional Review Board (Comité Nacional de Ética de la Investigación) and registered in the ClinicalTrials.gov database (NCT02034188). The study sponsor was Translational Biosciences. All treatments were administered at the Stem Cell Institute in the Republic of Panama, under Protocol Number TBS-UCMSC-001. Safety was defined as absence of treatment-associated adverse events at 1, 3 months, and 1 year post treatment. Efficacy was assessed with traditional MS evaluation instruments and a quality of life questionnaire at follow-up intervals, as detailed in the “Treatment protocol” section.

Subjects were enrolled under the following criteria: men or non-pregnant women ages 18–55 diagnosed according to revised McDonald criteria [24] for clinically-defined MS; an EDSS score of 2.0–7.0 assessed at least 3 months after the last acute attack of MS; willingness to keep a weekly diary and undergo observation for 1 year, and provision of documented health insurance in their home country. Enrolled subjects were not required to refrain from taking other medications or supplements prior to study entry.

Subjects were excluded if they presented active proliferative retinopathy, poorly controlled diabetes mellitus (glycosylated hemoglobin HbA1C > 8.5%), renal insufficiency (Creatinine > 2.5 mg/dL) or failure, infection (white blood cell count of > 15,000 K/cumm and/or temperature > 38 °C), history of organ transplant, previous or active malignancy, or cardiovascular conditions. All participants provided written informed consent before study participation.

### Treatment protocol

Complete medical history, medication history, and list of concomitant medications were collected from all subjects prior to any treatment. Subjects also underwent a complete physical examination, vital signs (heart rate, respiratory rate, temperature, and systolic and diastolic blood pressure), a 12-lead electrocardiogram (ECG), and laboratory testing (complete blood count, serum chemistry) at baseline. MS diagnosis was confirmed according to the revised McDonald criteria. Enrolled subjects received 140 × 10^6^ UCMSC intravenously over the course of seven visits (20 × 10^6^ UCMSC/day) separated by 1–4 days. At each treatment visit, subjects were assessed for any adverse events experienced since their last visit, received a physical examination (vital signs pre- and post- infusion), and were reviewed for adverse events throughout the visit.

Follow-up visits, scheduled at 1, 3 month and 1 year post-treatment, could take place at either the Stem Cell Institute or near the subjects’ place of residence, overseen by a licensed medical professional. At all follow-up visits, any adverse events experienced since last visit were reviewed, and subjects received a physical examination, laboratory tests and a 12-lead ECG. Any concomitant medications were reviewed.

Adverse events were reported in terms of their severity (mild, moderate, severe), relatedness (definitely, probably, possibly, not likely and unrelated), action taken (none, adjustment, interruption or discontinuation of treatment dosage), medications or therapy taken (drug therapy, non-drug therapy, or none), and outcome (not recovered/resolved, recovered/resolved, recovering/resolving, recovered/resolved with sequelae, fatal and unknown). Efficacy parameters were assessed at baseline, at 1 month, and at 1 year, and included time point measures for the Kurtzke Expanded Disability Status Scale (EDSS), the Scripps Neurological Rating Scale (SNRS), the Nine-Hole Peg Test (9HPT), the 25-Foot Walk Test (25FWT), and the RAND Short Form-36 (SF-36) quality of life (QOL) questionnaire.

Gadolinium-enhanced MRI scans of the brain and cervical spinal cord were taken at baseline and 1 year after treatment, and were examined by a single independent radiologist blinded to the intervention.

### UCMSC preparation and culture

UCMSC in this study were produced by MediStem Panama Inc. UCMSC were isolated from afterbirth tissue obtained after full-term, healthy births, donated by consenting mothers. After screening for infection and contamination, UCMSC were obtained from enzymatic digestion of Wharton’s Jelly tissue after a primary culture process at 37 °C, 5% CO2 during 24 h. Cells were expanded using alpha-MEM (Gibco Life Technologies, Grand Island, NY), supplemented with 10% FBS (USFDA approved, Gibco Life Technologies, Grand Island, NY), and 4 mM GlutaMax (Gibco Life Technologies, Grand Island, NY) in triple flasks under normoxic conditions. Cells were assessed between passages two and three for meeting MSC criteria and absence of contamination. Each enzymatic digestion step was considered to be a passage. Cells were harvested after 5 passages (3–4 weeks after initiation of primary culture). MediStem Panama used the minimal criteria established by the Mesenchymal and Tissue Stem Cell Committee of International Society for Cellular Therapy [25]. Each lot was tested for sterility (fungus, mycoplasma, aerobes and anaerobes), endotoxin level below 3 EU/ml, and viability after thawing higher than 75%. The approved cells expressed surface molecules CD105, CD73 and CD90, and lacked expression of CD45 and CD34, as determined by flow cytometry. UCMSC were also tested for differentiation into adipocytes, chondroblasts, and osteoblasts in vitro using StemPro^®^ media, and stained with oil red, alcian blue and alizarin red, respectively. Approved cells were suspended in a dextrose and saline solution for subsequent administration.

### Statistical analysis

SYSTAT version 13.1 (SYSTAT Software Inc., San Jose, CA, USA) was used to analyze the data. Differences between baseline and follow-up scores were examined using paired t-tests, with Bonferroni and Dunn-Sidak corrections where applicable. A p-value of p < 0.05 was considered significant.

## Results

Twenty subjects with MS provided informed consent and were enrolled into this feasibility study from October 10, 2014 to February 18, 2015. Mean age of enrollees was 41.15 (SD = 9.29) years; 60% (12/20) were female (Table 1). Enrolled subjects were of multiple international origins, including the Republic of Panama. The mean disease duration of enrollees was 7.7 years. Fifteen subjects (75%) had a diagnosis of relapsing–remitting MS, four (20%) with primary progressive MS, and one (5%) with secondary progressive MS. Eleven subjects (55%) required ambulation assistance (wheelchair, walker, or cane) at baseline. Five subjects (25%) did not take any MS-specific medication over the course of the study, 10 (50%) continued taking their usual MS medications, one (5%) began using MS-specific medication during follow-up, and four (20%) reduced their intake of MS-specific medication during follow-up.

**Table 1:** Demographics of subjects in the study

Demographic	N = 20
Age (years)	
Mean (SD)	41.15 (9.29)
(Range)	(24–55)
Gender	
Male	40% (8/20)
Female	60% (12/20)
Diagnosis	
Relapsing remitting MS	75% (15/20)
Primary progressive MS	20% (4/20)
Secondary progressive MS	5% (1/20)
Disease duration (years)	
Mean = 7.7	
< 3	25% (5/20)
4–6	25% (5/20)
7–9	30% (6/20)
10–12	10% (2/20)
16–18	5% (1/20)
19–21	5% (1/20)
Ambulation status	
Wheelchair	55% (11/20)
Walker	20% (4/20)
Bilateral cane	5% (1/20)
Unilateral cane	20% (4/20)
No assistance	45% (9/20)
Origin	
White/Caucasian	65% (13/20)
African Descent	10% (2/20)
Middle Eastern	5% (1/20)
Other (1 each Hispanic, Brazilian, Panamanian, and multiracial: Hispanic/White/Native American)	20% (4/20)

All subjects received all of the infusions specified by the treatment protocol, and attended the 1- and 3-month visits. Nineteen subjects were followed for 1 year: the 1-year visit was completed for 17/20 subjects, two subjects partially completed the 1-year requirements. One subject was lost to follow-up.

All subjects survived the study, and there were no reported serious adverse events (AEs). None of the reported AEs (Table 2) required adjustment, interruption or discontinuation of treatments. There were six moderate AEs, and 66 mild AEs. No AEs were ongoing at the 1-year follow-up visit. No AEs were classified as definitely related to study treatment. The most commonly reported AEs were headache (18 mild and one moderate), and fatigue (19 mild), classified as possibly related to treatment.

**Table 2:** Reported adverse events

Event	Count	Severity			Relatedness				
		Mild	Moderate	Severe	Not related	Not likely	Possibly	Probably	Definitely
Headache	19	18	1	0	1	1	16	1	0
Fatigue	19	19	0	0	0	2	17	0	0
Cardiovascular	8	8	0	0	2	2	4	0	0
Injury/accident	8	6	2	0	8	0	0	0	0
Gastrointestinal	4	2	2	0	1	2	1	0	0
Musculoskeletal	4	4	0	0	1	3	0	0	0
Infection	3	3	0	0	2	0	1	0	0
Feelings/sensations	3	3	0	0	0	2	1	0	0
Dizziness	2	2	0	0	1	1	0	0	0
Gynecological	1	0	1	0	1	0	0	0	0
Skin disorder	1	1	0	0	0	1	0	0	0
Total	72	66	6	0	17	14	40	1	0

**Fig. 1:**
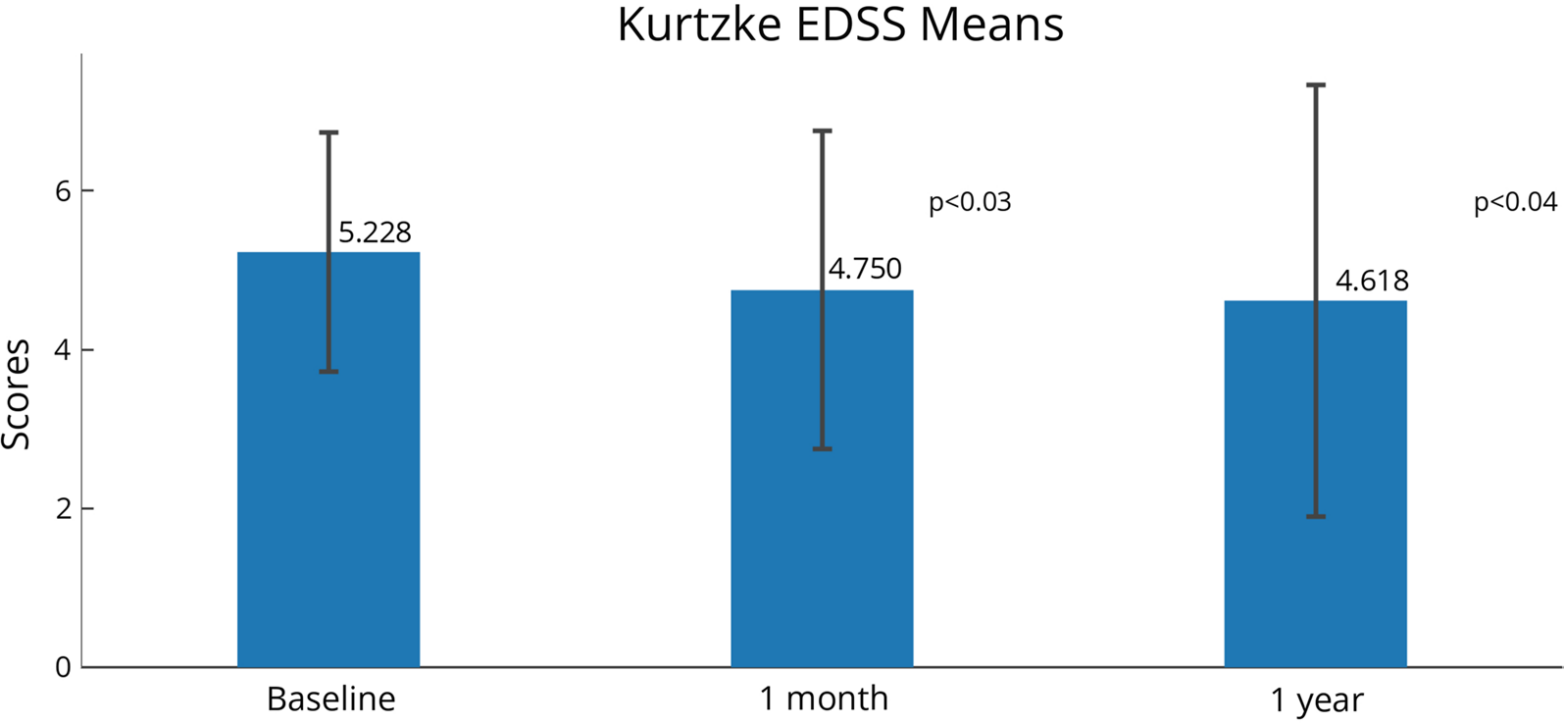
Kurtzke Expanded Disability Status Scale (EDSS) mean scores. Possible scores range from 0 (no disability) to 10 (death resulting from MS complications). Scores from 1.0 to 4.5 indicate an ability to walk without any aid, and scores from 5.0 to 9.5 indicate an impairment to walking. N = 20 at 1 month (same as baseline), N = 17 at 1 year. Statistically significant changes between time points are indicated with their p-values. Error bars represent standard deviations

EDSS scores were recorded at baseline and 1 month post-treatment for all subjects; scores at 1 year were available for 17 of 20 subjects (Fig. 1). At baseline, EDSS scores ranged from 2.5–7.0, with a mean score of 5.23 (SD = 1.50). At 1 month, the mean score decreased to 4.75 (SD = 2.00), a mean reduction of 0.48 (SD = 0.85) or about one category. Scores reduced further at the 1-year time point, to 4.62 (SD = 2.72), a mean reduction of 0.68 (SD = 1.49), a little more than one category. Differences were statistically significant at 1 month (p < 0.03), and 1 year (p < 0.04) when compared to baseline.

**Fig. 2:**
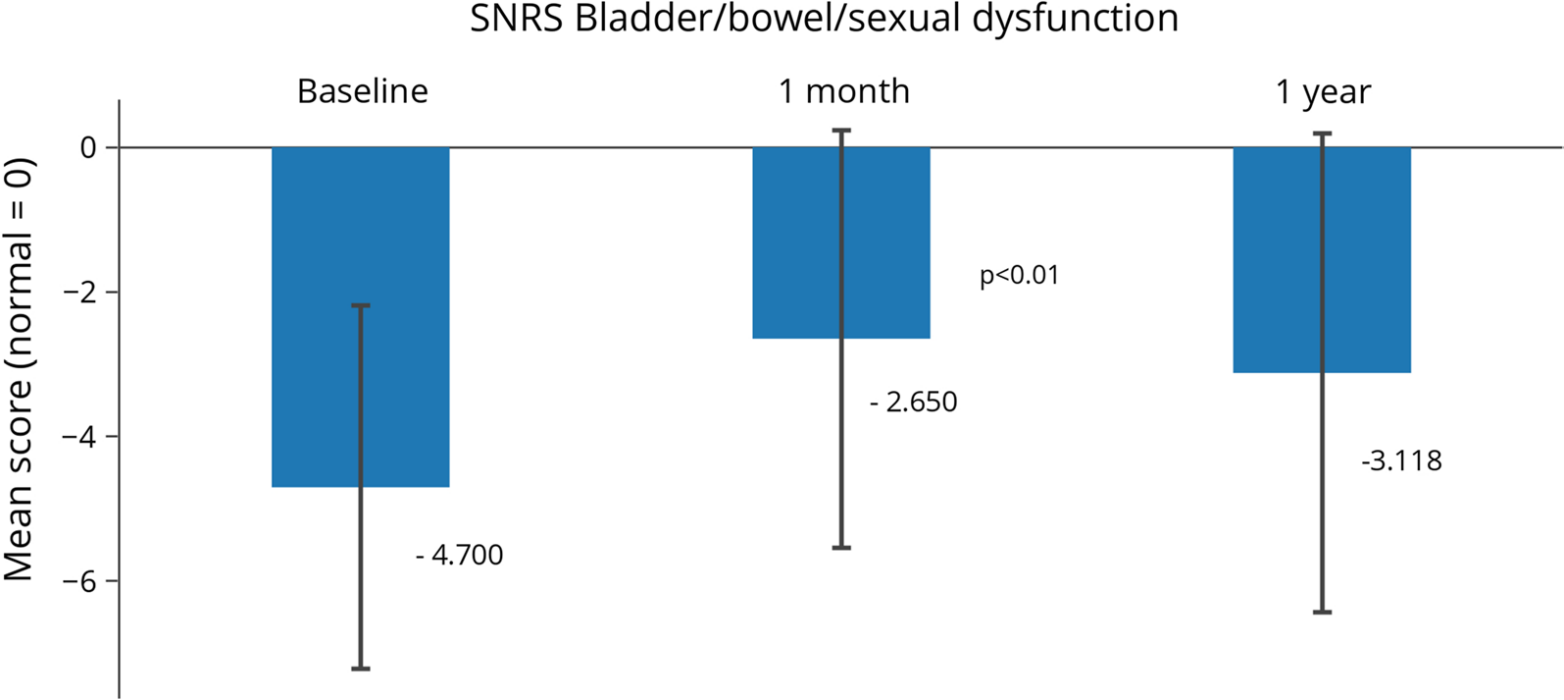
Scripps Neurological Rating Scale (SNRS) scores. Bladder, bowel and sexual dysfunction SNRS scores. The normal score is 0. A higher score on the SNRS indicates a higher level of neurological functioning, with possible scores ranging from − 10 to 100. N = 20 at 1 month (same as baseline), N = 17 at 1 year. Statistically significant changes between time points are indicated with their p-values

SNRS scores were recorded at baseline and 1 month post-treatment for all subjects; scores at 1 year were recorded for 17 of 20 subjects (Table 3). The SNRS total mean score rose slightly from 75.0 (SD = 11.9) at baseline to 75.5 (SD = 16.5) at 1 month post-treatment, and decreased to 73.5 (SD = 19.5, N = 17) 1 year post-treatment. A statistically significant improvement (p < 0.01) was seen for the bladder/bowel/sex dysfunction category at 1 month from the baseline assessment (Fig. 2). A statistically significant worsening was seen in the study group in four categories compared to baseline: visual acuity at 1 month (p < 0.02) and 1 year (p < 0.03), right upper extremity motor function at 1 year (p < 0.02), right lower extremity sensory function at 1 month (p < 0.03) and 1 year (p < 0.01), and left lower extremity sensory function at 1 month (p < 0.01).

**Table 3:** Scripps neurological rating system (SRNS) component scores

SNRS component	Baseline	1 month	1 year
Mentation and mood (normal = 10)	8.4 (2.28)	9.4 (1.57)	9.3 (1.69)
Visual acuity (normal = 5)*	4.9 (0.45)	4.2* (1.35)	4.2* (1.24)
Visual fields (normal = 6)	5.7 (0.73)	5.6 (1.05)	5.6 (0.79)
Eye movements (normal = 5)	4.8 (0.62)	4.8 (0.62)	4.8 (0.66)
Nystagmus (normal = 5)	5.0 (0.00)	4.8 (0.62)	4.9 (0.49)
Lower cranial nerves (normal = 5)	5.0 (0.00)	4.9 (0.45)	5.0 (0.0)
Motor function R upper extremity (normal = 5)*	4.8 (0.62)	4.5 (0.89)	4.1* (1.44)
Motor function L upper extremity (normal = 5)	4.3 (0.98)	4.4 (1.14)	4.1 (1.44)
Motor function R lower extremity (normal = 5)	3.2 (1.66)	2.6 (1.82)	2.4 (1.91)
Motor function L lower extremity (normal = 5)	2.7 (1.81)	2.7 (2.03)	2.7 (2.23)
DTR upper extremity (normal = 4)	3.6 (0.95)	3.3 (1.22)	3.6 (0.79)
DTR lower extremity (normal = 4)	2.4 (1.35)	2.1 (1.62)	2.3 (1.61)
Babinski sign L side (absent = 2)	0.6 (0.94)	0.9 (1.02)	0.7 (0.99)
Babinski sign R side (absent = 2)	0.8 (1.01)	0.8 (1.01)	0.5 (0.87)
Sensory R upper extremity (normal = 3)	3.0 (0.00)	2.7 (0.47)	2.8 (0.56)
Sensory L upper extremity (normal = 3)	3.0 (0.00)	2.6 (0.50)	2.8 (0.56)
Sensory R lower extremity (normal = 3)*	2.9 (0.31)	2.5* (0.51)	2.5* (0.72)
Sensory L lower extremity (normal = 3)	2.9 (0.31)	2.5* (0.51)	2.5 (0.80)
Cerebellar signs upper extremity (normal = 5)	4.0 (1.52)	4.0 (1.52)	4.1 (1.60)
Cerebellar signs lower extremity (normal = 5)	2.9 (1.46)	3.6 (1.87)	3.4 (1.77)
Gait trunk and balance (normal = 10)	5.1 (2.74)	5.6 (3.17)	4.6 (3.69)
Bladder, bowel, and/or sexual dysfunction (normal = 0)*	− 4.7 (2.52)	− 2.7* (2.89)	− 3.1 (3.31)
Total SNRS	75.0 (11.90)	75.5 (16.54)	73.5 (19.52)

*Statistically significant change, p < 0.05

**Fig. 3:**
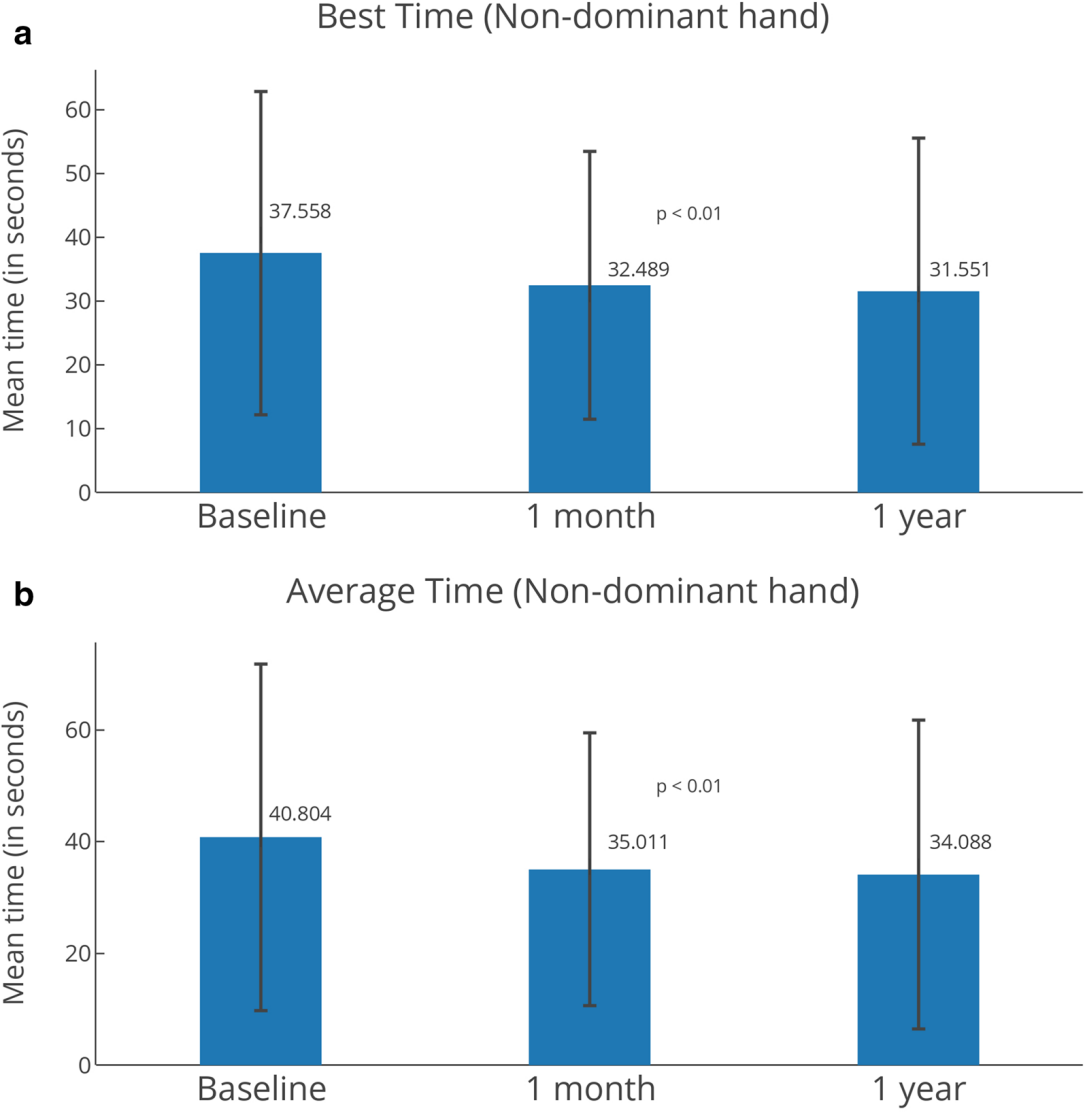
Nine Hole Peg Test (9HPT) for non-dominant hand. Best (**a**) and average (**b**) times for non-dominant hand. The 9HPT is an evaluation of arm/upper extremity functionality or disability. A reduction in the test time from the reference time point signifies an improvement of upper extremity function. N = 20 at 1 month (same as baseline), N = 19 at 1 year. Statistically significant changes between time points are indicated with their p-values. Error bars represent standard deviations

The 9HPT was recorded for all subjects at baseline and 1 month post-treatment; scores were available for 19 of 20 subjects at 1 year (one lost to follow-up). Overall, subjects saw improvements in their scores. Statistically significant improvements from baseline were seen in non-dominant hand scores for both best (p < 0.01) and average (p < 0.02) times at the 1-month assessment (Fig. 3). The best score for the non-dominant hand was 37.56 s at baseline, 32.49 at 1 month (5.1 s improvement), and 31.55 at 1 year (6.0 s improvement). The average mean score for the non-dominant hand was 40.80 s at baseline, 35.01 at 1 month (a 5.8 s improvement), and 34.09 (a 6.7 s improvement).

For the 25FWT, subjects were categorized into one of four categories, based on their need for assistance to complete the test (in order of increasing need): no assistance; cane; walker, and wheelchair. While scoring guidance for the 25FWT directs to use the average of the two completed trials, the minimum time was also analyzed to enable inclusion of any/all subject(s) who completed at least one full trial. At baseline, all 20 subjects were available for the 25FWT, and 19 subjects were available at 1 month (one did not take the test). At 1 year, results were recorded for 17 subjects (two did not take the test; and one was lost to follow up). Some subjects were unable to complete one or both trials due to ambulation status (two were wheelchair-bound at baseline, one at the 1-month and at 1-year follow-ups), or fatigue (one subject was fatigued at the 1-year follow-up).

**Fig. 4:**
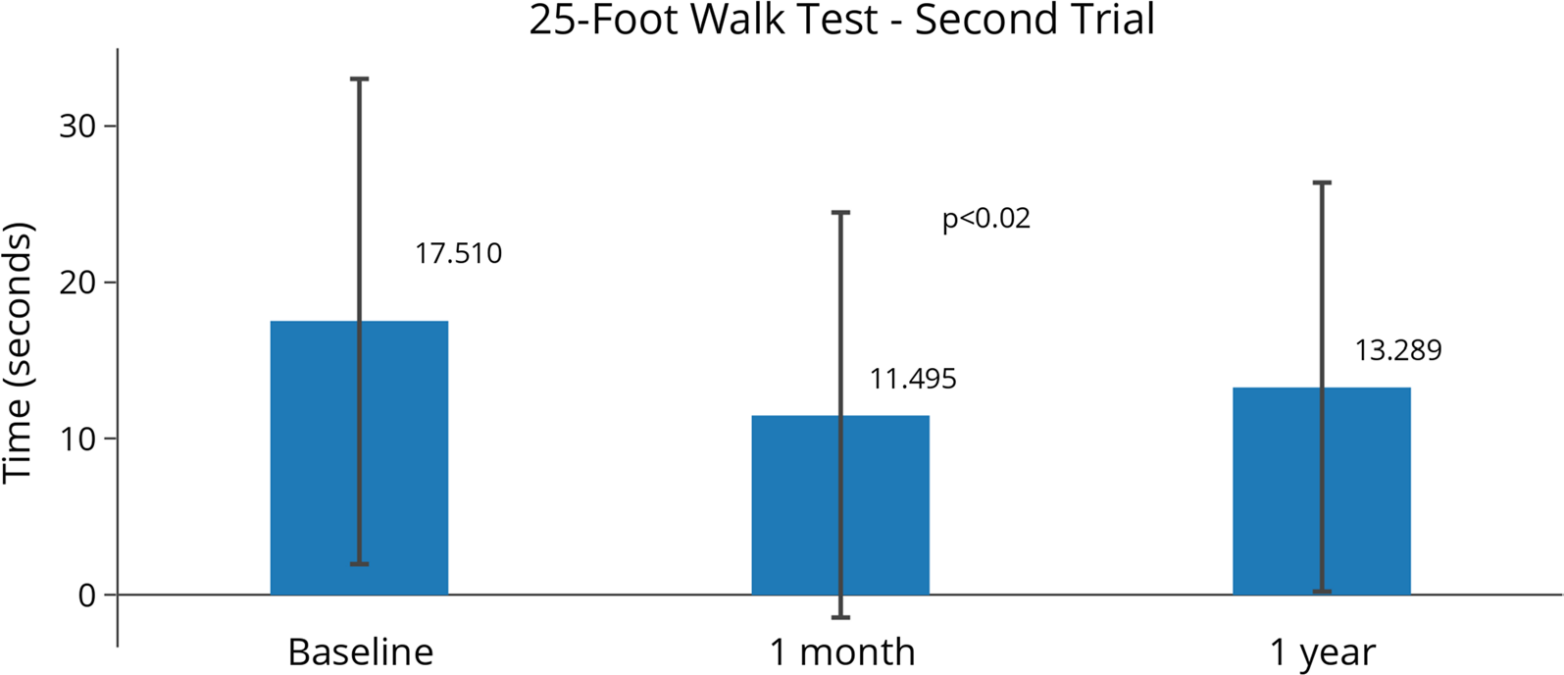
25 Foot Walk Test (25FWT) 2nd trial scores. Subjects were asked to perform two trials of a 25-Foot Walk. Not all subjects completed both trials, or performed these tests at all time points. In this figure N = 12, and statistically significant changes between time points are indicated with their p-values. Error bars represent standard deviations

At baseline, average time for the 25FWT Trial 1 was 17.43 s and 17.51 s for Trial 2 (Table 4). Overall mean time was 17.47 s, and average minimum time was 15.85 s. One month after treatment, average walk time for Trial 2 improved by 6.01 s, but average time for Trial 1 took longer than baseline, as did overall mean time, and average minimum time. At 1 year, all walk times were reduced when compared to baseline. Since not all subjects completed both trials of the 25FWT at a given time point, and not all subjects completed all follow-ups, statistical comparisons between baseline and follow-up scores could not include all subjects. Twelve subjects completed Trial 2 of the 25FWT at the three follow-up time points (baseline, 1 month and 1 year), with a statistically significant reduction (p < 0.02) in walk times found between baseline and 1 month (Fig. 4).

**Table 4:** 25-Foot Walk Test times

	Trial 1—average time	Trial 2—average time	Overall mean time	Average minimum time
Baseline	17.43 (N = 18)	17.51 (N = 18)	17.47 (N = 18)	15.85 (N = 18)
1 month	29.19 (N = 18)	11.50 (N = 13)	28.63 (N = 18)	28.01 (N = 18)
1 year	16.18 (N = 15)	13.29 (N = 13)	15.70 (N = 15)	14.71 (N = 15)

Some subjects changed ambulatory status at the time of the 25FWT at the 1-month visit: two subjects reduced their dependence upon assistive devices (one from wheelchair to walker, one from walker to cane); one became more dependent (from no assistance to a cane). At the 1-year visit, two subjects reduced their dependence upon assistance devices from baseline to 1-year visit (one from wheelchair to walker, one from walker to cane to no assistance); two increased dependency (one from unilateral cane to walker, one from no assistance to cane). One of the subjects, wheelchair-bound at baseline and thus unable to complete the test then, was able to complete it at the 1-month and 1-year follow-ups.

**Fig. 5:**
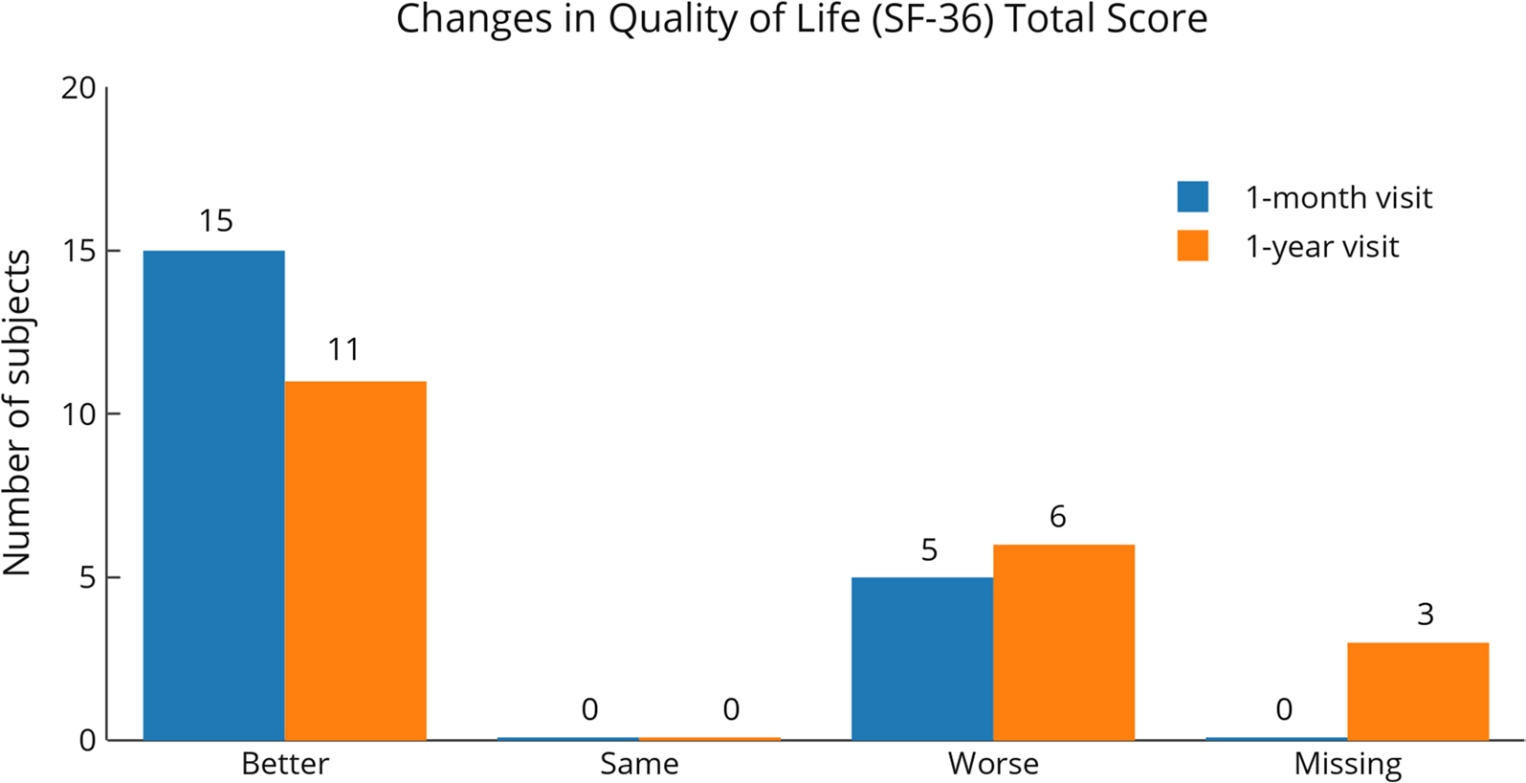
RAND SF-36 quality of life scores compared to baseline. Scores for the RAND SF-36 question capture health change. Scores were compared with baseline at the 1-month and 1-year visits. N = 20 at 1 month (same as baseline), N = 17 at 1 year

Scores for the RAND SF-36 test were recorded for all subjects at baseline and 1 month, and for 17 of 20 subjects at 1 year post-treatment. Overall, subjects reported improvements in their health during the study. Total QOL scores increased over baseline for 15 subjects at the 1 month follow up visit, while only five decreased. At 1 year, total QOL score improved over baseline for 11 subjects, while six decreased (Fig. 5). At 1 month and 1 year, more than half of the subjects reported their condition as better or the same on all scale scores (Table 5). At 1 month, more than 50% of subjects reported improvements on five of the eight scale scores (role limitations-physical, energy/fatigue, emotional well-being, social functioning, and general health), as well as health change; 45% (9/20) of subjects reported improvements in the pain scale, and 35% (7/20) of subjects reported improvements in their physical functioning and role limitations-emotional. At 1 year, more than half of subjects reported feeling better in five of the eight scales (physical functioning, role limitations-physical, energy/fatigue, emotional wellbeing, and general health), as well as health change; 47% (8/17) reported improvements in their social functioning, 35% (6/17) in pain scale, and 29% (5/17) in role limitations-emotional.

**Table 5:** RAND SF-36 changes in scores

	Baseline to 1 month (N = 20)			Baseline to 1 year (N = 17)		
	Better	Same	Worse	Better	Same	Worse
Physical functioning	7 (35%)	4 (20%)	9 (45%)	10 (59%)	1 (6%)	6 (35%)
Role limitations—physical^a^	13 (65%)	6 (30%)	1 (5%)	11 (65%)	4 (24%)	2 (12%)
Role limitations—emotional	7 (35%)	10 (50%)	3 (15%)	5 (29%)	9 (53%)	3 (18%)
Energy/fatigue^b^	16 (80%)	1 (5%)	3 (15%)	11 (65%)	0 (0%)	6 (35%)
Emotional well-being	13 (65%)	2 (10%)	5 (25%)	10 (59%)	2 (12%)	5 (29%)
Social functioning	12 (60%)	2 (10%)	6 (30%)	8 (47%)	1 (6%)	8 (47%)
Pain	9 (45%)	5 (25%)	6 (30%)	6 (35%)	8 (47%)	3 (18%)
General health	11 (55%)	1 (5%)	8 (40%)	9 (53%)	2 (12%)	6 (35%)
Health change^a^	13 (65%)	6 (30%)	1 (5%)	11 (65%)	4 (24%)	2 (12%)

^a^Differences between baseline and 1-month and 1-year scores statistically significant (p < 0.03)

^b^Differences between baseline and 1-month scores statistically significant (p < 0.001)

**Fig. 6:**
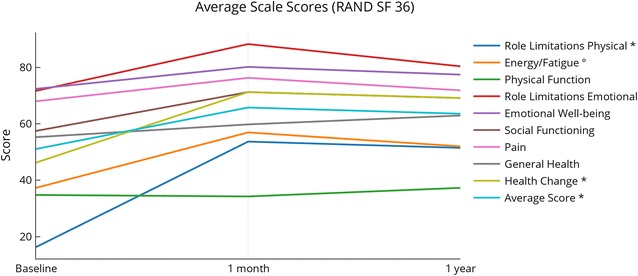
Changes in RAND SF-36 component scores. Changes in scores for all the RAND SF-36 categories. Asterisk denotes statistically significant changes (p < 0.05) both at 1 month and 1 year post-treatment; degree denotes statistically significant change (p < 0.05) at 1 month post-treatment

Statistically significant changes from baseline occurred at both 1-month and 1-year assessments for the RAND SF-36 role limitations-physical (p < 0.002 and p < 0.03, respectively) and health change (p < 0.004 and p < 0.02, respectively) categories. A statistically significant change occurred from baseline at 1 month in the energy category (p < 0.006) and the average score (p < 0.001). Bonferroni corrections retained statistically significant differences at 1 month in these four categories (p < 0.02). Most categories in the RAND SF-36 showed improvement over the baseline at 1 month and 1-year evaluations, with the exception of the 1-month physical function score. Most categories scored highest at the 1-month evaluation with the exception of the physical function category (Fig. 6).

**Fig. 7:**
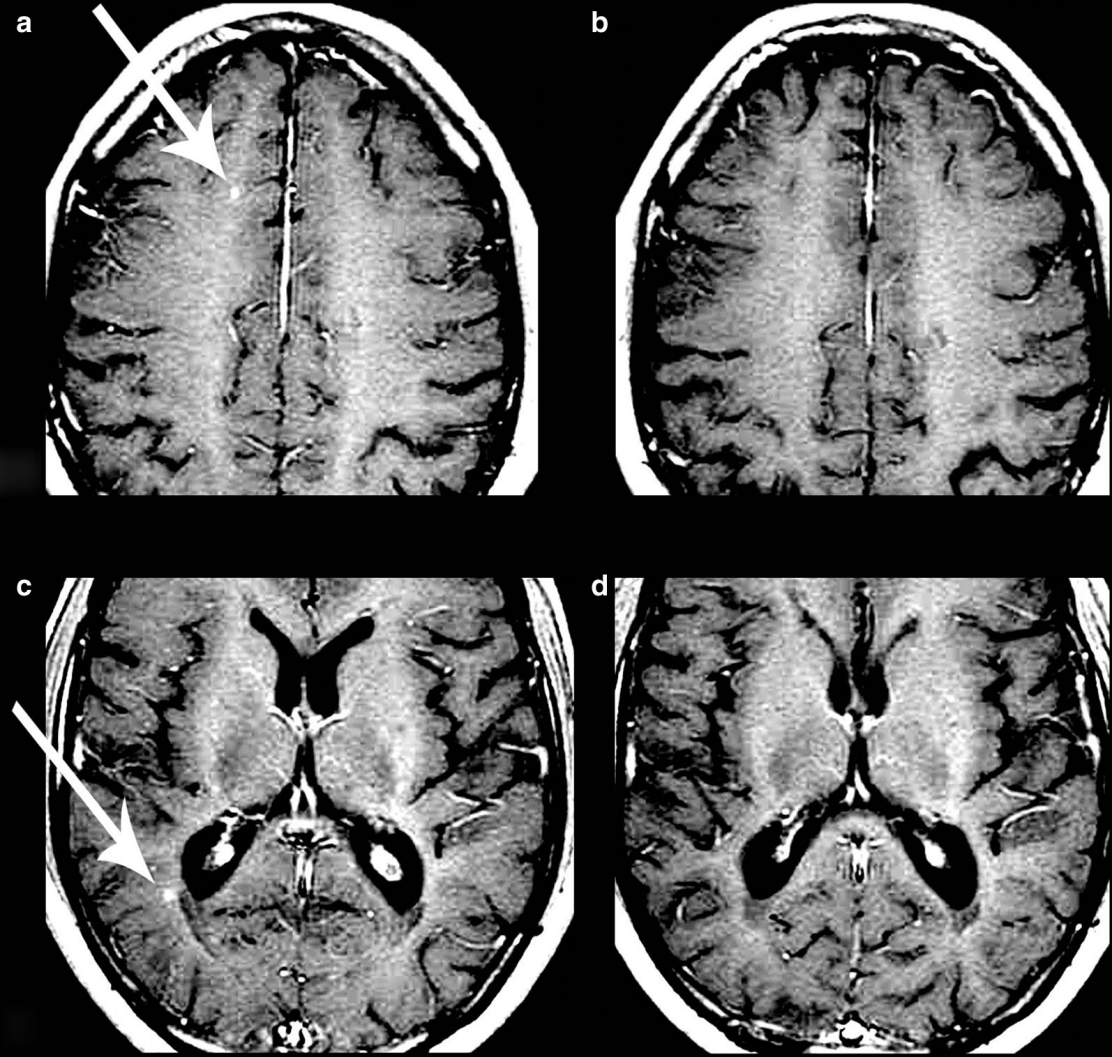
Changes in magnetic resonance imaging (MRI) before and after treatment. Gadolinium-enhanced MRI scans of the brain for one subject before (**a** and **c**) and after (**b** and **d**) treatment. Lesions of interest are indicated by a white arrow. **b** Interval resolution of a lesion in the right frontal juxtacortical white matter (**a**). **d** Interval resolution of a lesion in the right periatrial white matter (**c**). Names and other personal information have been edited out of the images

Pre- and post-treatment MRIs from the brain and the cervical spinal cord were reviewed qualitatively for every subject. MRI was unavailable at the 1-year follow-up for two subjects. Not all subjects had the second MRI taken at exactly 1 year after treatment; there was some variation in completion of the second MRI, up to 4 months after the 1-year mark. No common themes were detected regarding number of lesions or disappearance of lesions. Of the 18 subjects that did complete MRI both at baseline and at the 1-year follow-up, 15 (83.3%) showed no disease progression or no new or active lesions. Two subjects (11.1%) showed progression in their lesions. One patient (5.6%) showed near complete resolution of the plaques in the brain, in previously noted areas of abnormal signal intensity consistent with demyelinating disease (Fig. 7).

## Discussion

The demographics of this study correspond to those of an expected MS population, where women are typically affected twice as frequently as men, and most patients are diagnosed between 20 and 40 years of age [26–29].

No Serious Adverse Events occurred in the study. One single Adverse Event (AE), headache, was noted as probably related to treatment. Of the AEs denoted as possibly related to treatment, most were headache or fatigue. Headache is a known complaint noted to occur during or just after MSC infusions, most of which quickly resolve [30]. Additionally, Foley et al demonstrated that patients with MS commonly experience headache [31]. Thus, headache is not unexpected at the time of or just after UCMSC infusions. Similarly, fatigue is one of the most common complaints [32]. Fatigue appears in all ages and phenotypes of MS [33–35] and is a primary determinant of poor QOL [35], affecting both physical and mental components independent of disability level [34]. Thus, although fatigue is a possibly related AE in this study, it is also a very common disability symptom of MS.

Enrolled subjects experienced an improvement in their symptoms, which was most notable at 1 month after treatment, and was sustained at 1 year in some cases (Table 6). The potential durable benefit of UCMSC at 1 month, and sustained in some measures to 1 year, is in stark contrast to current MS drug therapies, which are required to be taken daily or weekly [36]. In addition, MS drugs are known to carry side effects [37] not seen after UCMSC infusions up to 1-year after treatment.

**Table 6:** Summary of efficacy assessment scores

Test	Baseline	1 month	1 year
EDSS mean scores	5.2	4.8*	4.5*
SNRS bladder/bowel/sexual dysfunction score (normal = 0)	− 4.7	− 2.7*	− 3.1
Nine Hole Peg Test—non-dominant hand average scores	35.1	30.2*	34.1
25-foot walk (second trial) times	17.5	11.5*	13.3
RAND SF-36 average scale	53.5	68.3***	63.5

* p < 0.05 when compared to baseline; *** p < 0.001 when compared to baseline

Previous studies using MSC treatment for MS have reported improvements in EDSS scores [19, 22, 23, 38, 39]. In our case, the statistically significant (p < 0.03) change in EDSS mean scores from baseline to 1 month reflects a change in disability category, which could translate into an improved ability to walk and work a full day with minimal, if any, assistance. Although other categories showed worsening typical of disease progression, the bladder/bowel/sexual dysfunction category of the SNRS showed statistically significant improvement at 1 month (p < 0.05). This finding may be encouraging in that up to 60% of patients with MS report sexual dysfunction problems [40] while over 50% experience bowel dysfunction and up to 75% will report bladder dysfunction [41].

Changes to ambulatory status from baseline classifications were noteworthy in this study for the 25FWT. Increasing disability (e.g., going from unassisted walking to a cane) is a typical disease progression in the MS population in general, but the reverse would not be expected. However, subjects in our study improved from wheelchair status to using a walker, and from walker status to requiring no assistance.

In general, QOL for patients with MS is diminished by physical, emotional, and cognitive symptoms and comorbidities [33, 42]. However, subjects enrolled in this study reported consistent improvements in their RAND 36 SF QOL in these areas, particularly at the 1-month evaluation (p < 0.001). Most categories improved at 1 month, and then slipped slightly at the 1 year evaluation period while still remaining improved over baseline, suggesting that treatment frequency greater than once annually could further improve the treatment subject’s QOL outlook.

Most subjects (83.3%) showed no disease progression or new lesions in their MRIs. The near complete resolution of the plaques of the brain in one patient (Fig. 7) is a particularly encouraging finding that should be further investigated by comparing it to similar MS cohorts in a standardized time period.

The small sample size is the most significant limitation of this study in that it may impact the statistical significance of the results. When subjects were lost to follow-up, the sample size was reduced; due to the nature of certain tests used to measure efficacy signals, failing to complete one part of the test often invalidated obtaining an average for the score. While this issue could have been avoided with larger recruitment numbers, we are still in the lower threshold of the 20–80 subjects recommended by FDA for early trials. Additionally, subjects with certain forms of MS may be less likely to develop new lesions, which may impact the findings of the reported MRI results. As this was primarily a safety and proof of concept trial, we did not require subjects to stop their usual medications, which could have a confounding impact on our findings. Poor medication adherence is frequently seen among patients with MS (usually because of cost, perceived lack of efficacy, or adverse effects) [43]; in our sample, 25% were not taking any MS-specific medication at the start of treatment. However, it is noteworthy that a further 20% felt well enough to reduce their intake. In any case, efficacy of UCMSC therapy for MS should be confirmed with larger, controlled, randomized trials.

## Conclusions

We have shown that the intravenous infusion of UCMSC over several days is safe in subjects with MS. Additionally, UCMSC infusions may hold benefits, since this small study group saw improvement in bladder, bowel, and sexual dysfunction, walking, upper extremity physical function, energy and fatigue, general perspective of a positive health change and improved quality of life, and MRI lesions. More clinical studies, particularly with a larger cohort, are needed to substantiate the specific benefits of UCMSC infusion as a potential MS therapy.
