# Correction to: Clinical feasibility of umbilical cord tissue-derived mesenchymal stem cells in the treatment of multiple sclerosis

**DOI:** 10.1186/s12967-021-02869-4

**Published:** 2021-05-10

**Authors:** Neil H. Riordan, Isabela Morales, Giselle Fernández, Nicole Allen, Neal E. Fearnot, Michael E. Leckrone, Dedra Jones Markovich, Darla Mansfield, Dorita Avila, Amit N. Patel, Santosh Kesari, Jorge Paz Rodriguez

**Affiliations:** 1 Stem Cell Institute, Panama City, Panama; 2 MediStem Panama Inc., Clayton, City of Knowledge, Panama City, Panama; 3 Cook Advanced Technologies, West Lafayette, IN USA; 4 Cook Medical Technologies, Bloomington, IN USA; 5 Department of Surgery, University of Miami School of Medicine, Miami, FL USA; 6 Department of Translational Neurosciences and Neurotherapeutics, John Wayne Cancer Institute and Pacifc Neuroscience Institute, Santa Monica, CA USA

## Correction to: J Transl Med (2018) 16:57 10.1186/s12967-018-1433-7

The original publication of this article [1] contained 2 errors in the funding and discussion section. In this correction article the incorrect and correct information is published for clarification.

The following sentence in the Funding section, **“The study sponsor had no role in the design of this study, its execution, analysis, interpretation of the data, writing, or decision to submit results.”** should read **“The study sponsor had a role in the design of this study, its execution, analysis** ***as JPR is a shareholder of Translational Biosciences, MediStem Panama, and the Stem Cell Institute."***

The following sentence in the Discussion last paragraph, **“The small sample size is the most significant limitation of this study in that it may impact the statistical significance of the results.”** in the limitations paragraph should read instead **“The small sample size is the most significant limitation of this study in that it may impact the statistical significance of the results,** ***when considering the suitability of a t-test on a small sample size.”***
